# A comprehensive review of the role of bone marrow biopsy and PET-CT in the evaluation of bone marrow involvement in adults newly diagnosed with DLBCL

**DOI:** 10.3389/fonc.2024.1301979

**Published:** 2024-03-21

**Authors:** Ruah Alyamany, Riad El Fakih, Ahmed Alnughmush, Abdulwahab Albabtain, Mohamed A. Kharfan-Dabaja, Mahmoud Aljurf

**Affiliations:** ^1^ Department of Hematology, Stem Cell Transplant and Cellular Therapy, King Faisal Specialist Hospital and Research Centre, Riyadh, Saudi Arabia; ^2^ Division of Hematology-Oncology, Blood and Marrow Transplantation Program, Mayo Clinic, Jacksonville, FL, United States

**Keywords:** diffuse large B-cell lymphoma, bone marrow involvement, bone marrow biopsy, positron emission tomography/computed tomography (PET-CT), lymphoma

## Abstract

Diffuse large B cell lymphoma (DLBCL) is one of the most prevalent subtypes of non-Hodgkin lymphoma (NHL) and is known for commonly infiltrating extra-nodal sites. The involvement of the bone marrow by lymphoma cells significantly impacts the staging, treatment, and prognosis among the extra-nodal sites in DLBCL. Bone marrow biopsy has been considered the standard diagnostic procedure for detecting bone marrow involvement. However, advancements in imaging techniques, such as positron emission tomography-computed tomography (PET-CT), have shown an improved ability to detect bone marrow involvement, making the need for bone marrow biopsy debatable. This review aims to emphasize the importance of bone marrow evaluation in adult patients newly diagnosed with DLBCL and suggest an optimal diagnostic approach to identify bone marrow involvement in these patients.

## 1 Introduction

Non-Hodgkin lymphoma (NHL) constitutes approximately 4% of annual cancer diagnoses. It ranks as the sixth most common cause of cancer and accounts for almost 6% of malignancy-related mortality in Europe and the United States (1–3). Within the spectrum of NHL, diffuse large B cell lymphoma (DLBCL) emerges as the most prevalent subtype, accounting for 30 – 40% of aggressive NHL cases in adults worldwide (3–7). The diagnosis of DLBCL depends on histological confirmation, complemented by clinical and radiological findings (2). DLBCL is recognized for its variable biological and clinical features, along with frequent extra-nodal site infiltration, which has a momentous impact on the staging and, consequently, management and prognosis (2, 7, 8). Accurate staging of DLBCL is crucial for optimal management, with the Ann Arbor staging system being one of the most recognized systems, initially relying on physical examination and bone marrow biopsy (2). Other staging systems used in NHL include the Lugano and LYRIC criteria (9, 10). The advancements in diagnostic medicine and the development of positron emission tomography and computed tomography (PET-CT) have revolutionized disease assessment and treatment response evaluation. PET-CT has significantly influenced the initial staging and subsequent disease re-assessment following therapy in DLBCL. Its impact has become increasingly evident since immunotherapy agents have become widely available and PET-specific response criteria have been developed (11). These advancements have increased the diagnostic and prognostic value of PET-CT in managing DLBCL. Despite the progression in imaging techniques, the value of PET-CT in detecting bone marrow involvement in DLBCL and its ability to replace bone marrow biopsy remains controversial (2).

The International Prognostic Index score (IPI score) has been widely accepted as a prognostication tool for risk-stratifying patients with DLBCL. In the pre-rituximab era, the IPI score had limitations in identifying higher-risk patients, which urged its revision by the National Comprehensive Cancer Network (NCCN) guidelines (12). This adjustment aimed to improve the ability of the IPI score to discriminate low-risk from high-risk patients, particularly in DLBCL patients treated with rituximab, with a focus on overall survival (OS) (**Table 1**) (13, 14).

**Table 1:** International Prognostic Index (IPI) Score Components (13).

Age	>60 years
Eastern Cooperative Oncology Group (ECOG) Performance status	More than or equal to 2
Ann Arbor stage	Stage III or IV
LDH	Elevated >1x normal level
Number of involved extra-nodal sites	More than or equal to 2 (Bone marrow, GI tract, Liver, Lung, CNS, Skin, Testes, Waldeyer’s ring)

## 2 Bone marrow involvement in DLBCL

In diffuse large B-cell lymphoma (DLBCL), the involvement of extra-nodal tissue often signifies a more advanced disease, correlating with poorer outcomes (2). Bone marrow (BM) involvement is reported in 10-30% of DLBCL cases, making it a critical aspect of the initial evaluation of DLBCL as it holds prognostic and therapeutic implications (6). Specifically, BM involvement has been linked to suboptimal prognosis in DLBCL patients with advanced Ann Arbor staging and higher IPI scores (3, 4, 7, 12, 15–17). In cases with limited stages, bone marrow involvement leads to upstaging and necessitates adjustments in the management plan (18). Individuals with BM involvement in DLBCL face an increased risk of primary refractory disease reported at a rate of 10-15%, a 20-30% chance of relapse (8), and, notably, as reported in one study, a higher incidence of rituximab infusion-related reactions (19). Consequently, a more aggressive therapeutic approach may be warranted for patients with bone marrow involvement. Notably, the standard iliac crest bone marrow biopsy demonstrates a limited ability to detect BM involvement in DLBCL, identifying only 27% of patients with confirmed bone marrow involvement (4, 7, 8, 20). The inclusion of other diagnostic techniques, including flow cytometry, enhances the biopsy’s sensitivity in detecting bone marrow involvement (2, 7). PET-CT frequently identifies bone marrow involvement in a focal or diffuse uptake distribution, while bone marrow biopsy relies on the morphological examination of slides, immunohistochemistry stains, and flow cytometry to detect lymphoma cells and specific clusters of differentiation (CD) markers (8).

## 3 Methods for bone marrow involvement detection

The two most commonly used techniques for determining bone marrow involvement in DLBCL are bone marrow biopsy and PET-CT scan.

### 3.1 Bone marrow aspirate and biopsy

The gold standard for assessing bone marrow involvement in lymphomas has been a random unilateral, occasionally bilateral, posterior iliac crest trephine bone marrow biopsy and aspirate. Previously, this procedure was a crucial part of the staging process (2–4, 8, 21). This procedure is usually performed blindly (i.e., not directed towards a lesion) at the bedside under local anesthesia and aseptic precautions. While there is no universally accepted definition for an adequate sample size, consensus suggests an acceptable range of 0.5 – 1 cm, but optimally 2 – 3 cm (8, 17, 22). Although bone marrow biopsy is considered relatively safe, it has its risks and limitations (**Table 2**) (2, 3, 8). Complication rates, as reported in one study, were approximately 0.07%, with bleeding, most frequently retroperitoneal hemorrhage, being a critical and severe complication, particularly associated with certain risk factors such as myeloproliferative neoplasms, platelet dysfunction, anticoagulation use, disseminated intravascular coagulation (DIC), renal impairment, and obesity (24–28). It is worth noting that the operator’s years of experience did not show a clear correlation with bleeding rates (27).

**Table 2:** Advantages and Disadvantages of Bone Marrow Biopsy.

Advantages	Disadvantages
• Aids in diagnosing DLBCL in those without extramedullary biopsy (23). • Can identify a low-volume marrow infiltrate or underlying indolent lymphoma and provide information on the presence of MDS features which can change treatment (21, 23).	• Invasive procedure (7, 16). • Bleeding (1, 8) • Pain (2, 7) • Anxiety* (2, 21) • Infections (skin and soft tissue, osteomyelitis) (2, 8, 17, 21) • Allergy and anaphylaxis (1, 8) • Fractures at site of biopsy, especially in patients with osteoporosis (1) • Time consuming** (1, 3, 23) • Limited view due to site-dependance, can miss patchy disease not involving the iliac crest (1, 3, 4, 7, 17) • Seeding of lymphoma cells into adjacent soft tissue (1) • Unpleasant experience (2) • Sampling error, leading to false negative results (2, 3) • Needle breakage (17) • Failure of procedure*** (8)

*Anxiety can lead to significant stress and ultimately lead to patients missing their appointments, which can further delay the results (1).

**Sample preparation is time-consuming; decalcification requires a long time, and analysis takes longer (3).

***Failure of procedure has been reported in 0.12% of cases (8).

The necessity of bone marrow aspirate and biopsy has recently been debatable for evaluating bone marrow involvement in DLBCL (16). Some experts continue to support the usage of bone marrow biopsy by citing studies showing that patients with histologically confirmed bone marrow involvement had inferior overall survival (OS), event-free survival (EFS), and progression-free survival (PFS) results (12, 14, 20). Furthermore, histological examination provides insight into the specific cell types involved, as bone marrow could be infiltrated by an unrelated lymphoma, such as indolent lymphoma, which is known as discordant bone marrow involvement (4). Histological examination of the bone marrow continues to be essential in patients with early-stage disease based on imaging, as it may significantly impact disease upstaging and subsequent treatment decisions (23).

### 3.2 Positron emission tomography and computed tomography

PET-CT, a radiological technique used in staging aggressive lymphomas, provides a comprehensive 3-dimensional whole-body image for evaluating cellular metabolic activity and function using radioactive agents (17). Several types of PET-CT use different tracers, including but not limited to fluorine-18-deoxyglucose, sodium fluoride, and oxygen-15 (29–33). The most commonly used radiopharmaceutical agent in oncology is Fluorine-18-deoxyglucose (18F-FDG), a glucose analog that facilitates the detection of metabolically active sites. Following the administration of the FDG agent, images are captured using a full-ring detector PET scanner combined with a multidetector helical CT machine. While PET combined with CT yields superior results compared to PET alone, the inclusion of CT introduces higher radiation exposure. However, low-dose (80 mAs) PET-CT options are available for staging, minimizing radiation exposure (34). Standardized uptake values (SUV), the most frequently used parameter, express the ratio of radioactivity concentration within the region of interest (ROI) to the decay-corrected amount of injected radio-labeled FDG (kBq) per patient’s weight (kg), are used to report metabolic activity, presented as kBq/ml (8, 35). SUV is corrected in patients of extreme body weights, using the lean body weight (SUL) in obese patients and body surface area in patients of small body weight (36). Other less commonly used parameters in the assessment of tumor volume and metabolic activity include metabolic tumor volume (MTV), total lesion glycolysis (TLG), and tumor-to-blood ratio (TBR) (37–39). PET-CT is considered the standard tool for lymphoma staging, exhibiting high sensitivity and specificity in identifying lymphoma activity sites, particularly in Hodgkin’s lymphoma and aggressive subtypes of NHL like DLBCL, owing to their heightened FDG avidity (2, 21, 40). Notably, PET-CT scans accurately identify both nodal and extra-nodal involvement, facilitating precise staging and prognosis quantification (4). This positions PET-CT as the preferred modality for pre-treatment disease assessment and post-therapy response evaluation (2). Achieving greater consistency in data is crucial for evaluating the efficacy of PET-CT in detecting bone marrow involvement, particularly in instances of discordant bone marrow involvement by a different lymphoma subtype (21). Across the majority of published literature, PET-CT demonstrates excellent precision in detecting concordant bone marrow involvement. In a study by Khan et al., PET-CT exhibited exceptional accuracy, with only two cases showing negative PET-CT results but positive bone marrow biopsy results, both consistent with low-volume disease (i.e., 10% large B cells) and already labeled as stage IV based on uptake elsewhere (4). Another study supports the accuracy of PET-CT in detecting bone marrow involvement, at least as effectively as bone marrow biopsy (1). PET-CT’s remarkable ability to detect bone marrow involvement is particularly evident in concordant cases with aggressive B-cell lymphomas like DLBCL, which usually shows increased FDG avidity and higher SUV on imaging with a high Deauville score, which is an ordinal 5-point scale that relies on the visual comparison between the glucose uptake of the tumor and that of the liver or mediastinum (34, 41–43). However, PET-CT’s accuracy weakens in discordant cases where the marrow may be involved by a lymphoma other than DLBCL (1, 40, 44). PET-CT surpasses CT with bone marrow biopsy in identifying occult lymphoma sites (40). For optimal results, PET-CT should be performed in adherence to standardized procedures and be interpreted by highly experienced radiologists and nuclear medicine specialist with focused training in PET-CT evaluation for lymphomas. Less experienced interpretation may lead to overanalysis of FDG uptake in the bone marrow and increased false-positive rates (7, 12, 17, 45). To enhance the accuracy of PET-CT and avoid incorrect results, a unified definition of bone marrow involvement is essential. Positive bone marrow involvement by PET-CT can be defined by several features:

1. The mean SUV max, measured by FDG uptake, should be higher than that of the liver, quantifying at more than 3.8 with a Deauville score of 4 or 5 (4, 7, 8, 15, 16, 46). Higher SUV is often associated with positive bone marrow involvement by lymphoma (4).
2. Bone marrow involvement should not be a contiguous spread from nearby disease involving soft tissue (4).
3. No anatomical changes should suggest an alternative underlying benign abnormality (4, 21).
4. Increased FDG activity at sites of previous bone marrow biopsy or fractures is considered negative (15).

Some references do not consider diffuse bone marrow uptake on PET-CT as positive for bone marrow involvement unless proven by histopathology review (12, 21).

Additional methods to confirm disease-related bone marrow uptake on PET-CT include:

1. Confirmation through MRI imaging on bone lesions (18, 40).
2. Repeat PET-CT after treatment to assess if uptake resolves with the resolution of involved nodal sites (18).
3. Tissue biopsy of the enhanced lesion (i.e., directed biopsy) (18, 40).

It is crucial to acknowledge that not all lesions on PET-CT can be confirmed by the mentioned methods (4). While PET-CT offers user-friendly convenience, it is not without its inherent limitations (**Table 3**).

**Table 3:** Advantages and Disadvantages of PET-CT.

Advantages	Disadvantages
• Non-invasive procedure (2, 40). • Ability to assess metabolic activity of lesions with the lymph nodes and in extra-nodal sites (12, 40). • Capacity to assess the entire marrow including patchy disease outside the iliac crest along with extra-medullary disease (2, 4, 8, 40). • It provides the benefit of continuous non-invasive monitoring in situations of uncertainty.	• Radiation exposure (40). • Patient needs to be fasting with controlled glucose levels prior to PET-CT (40). • Areas with physiologically high FDG uptake such as the brain, heart, digestive and urinary collecting systems can mask underlying pathological uptake (40). • Receiving steroids, chemotherapy or radiation exposure prior to PET-CT can interfere with the imaging accuracy* (4, 40). • Age can influence the pattern of bone marrow FDG activity** (3, 47, 48). • Lacks histological confirmation of disease involvement (3). • Not all FDG avid lesions are related to lymphomatous involvement, i.e., false positive results*** (3). • PET-CT can miss bone marrow involvement in low-volume diseases or in cases of discordant bone marrow involvement with indolent lymphomas (4, 7, 12, 20, 49, 50). • Experience needed for accurate evaluation of uptake and interpretation regarding bone marrow involvement (12). • Using different types of scanners could result in different results **** (40).

*The optimal timing of PET-CT following the exposure to chemotherapy and radiation therapy to minimize false positive or inaccurate results is three weeks after chemotherapy and 8-12 weeks following radiation exposure (40).

**Age’s effect on the bone marrow activity on PET-CT is controversial; there is not enough evidence to conclude on this point (3).

***False positive PET-CT can be seen with processes involving increased glucose metabolism and glycolysis, such as inflammation, infections, and granulomatosis diseases. Other causes of false positive PET-CT include bone marrow hyperplasia, such as in patients who use growth factors (e.g., Granulocyte stimulating factors (GCSF), TPO agonists and EPO agonists) or post-chemotherapy or in patients with cytopenia leading to bone marrow hyperactivity to compensate for the low cell counts (40). Some experienced radiologists are able to differentiate based on the FDG uptake pattern and imaging if the bone marrow’s uptake is reactive (3, 4, 7, 17).

****Using different scanners can lead to inaccurate results, especially when the scanner’s resolution is not optimal, leading to missing lesions below the scanner’s resolution (40).

PET-CT exhibits an enhanced utility in the context of DLBCL therapeutic advancements, particularly with the increased use of immunotherapy. The use of PET-CT in DLBCL has added significant value in initial diagnosis, staging, assessment of extra-nodal uptake, response assessment, and has further extended to the ability to detect signs of toxicities related to immunotherapy, such as inflammatory reactions, reactive changes, and tumor flare reactions (51). PET-CT has also had an important role in assessment of response to therapy, using several metrics to distinguish between different types of response, pseudo-progression, and progression (51). These facilities of PET-CT reinforce its diagnostic capabilities in aggressive lymphomas.

## 4 Patterns of bone marrow involvement in DLBCL

Bone marrow involvement can be categorized based on the detection method, the infiltrative cell type, and the imaging distribution.

### 4.1 Classification based on infiltrative cell type

Histopathological findings play a crucial role in classifying bone marrow involvement into two main categories: concordant bone marrow involvement, where features align with DLBCL characteristics in the bone marrow, and discordant bone marrow involvement, which exhibits features of lymphomas other than DLBCL, including indolent lymphomas such as follicular lymphoma, marginal zone lymphoma, and mantle cell lymphoma (20, 21). The majority of cases presenting bone marrow involvement fall under the concordant category (20). Discordant bone marrow involvement can be further classified into clonally-related and clonally-unrelated lymphomas based on immunoglobulin gene rearrangements (20). This distinction aids in discriminating between actual discordance, where two different pathologies coexist, and transformation of indolent lymphoma to DLBCL. When discordant bone marrow lymphoma cells and nodal DLBCL cells exhibit clonal similarity, there is an increased probability that the DLBCL arises as a transformation from the original indolent lymphoma (20). Notably, most cases of DLBCL with discordant bone marrow involvement that experience disease relapse show progression of the more aggressive DLBCL rather than transformation of their indolent lymphoma (discordant bone marrow involvement) (13). Prognostic data on biopsy-proven concordant bone marrow involvement is scattered. Sehn et al. reported that cases with concordant bone marrow involvement, histologically proven DLBCL, demonstrated an inferior outcome in terms of OS and PFS (14). Discordant bone marrow involvement was associated with lower PFS, supporting the presumed prognosis based on the IPI score in these patients (14). Additional studies supported the findings of poor OS in patients with concordant bone marrow involvement (52–54). It is worth noting that discordant bone marrow involvement did not independently correlate with inferior PFS or OS (16, 52–54).

### 4.2 Classification based on uptake distribution on imaging

**Figure 1:**
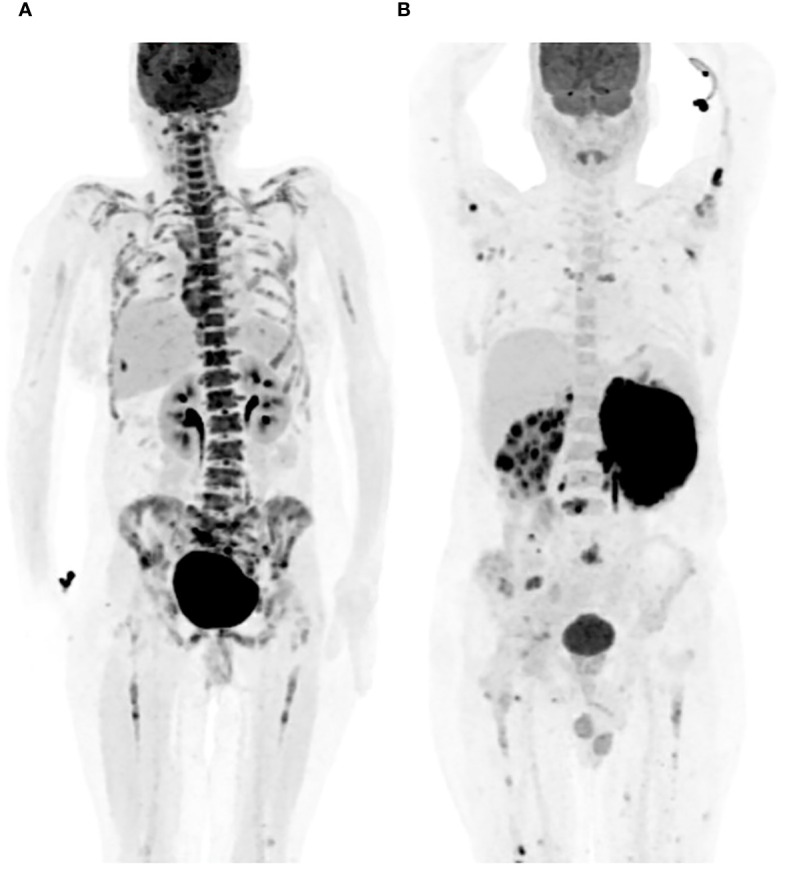
Patterns of bone marrow involvement on PET-CT. **(A)** Diffuse bone marrow FDG uptake on PET-CT. **(B)** Multifocal, scattered, bone marrow FDG uptake on PET-CT.

Bone marrow uptake observed in PET-CT scans manifests in various patterns, including focal, diffuse, or a combination of both, with focal areas exhibiting higher FDG uptake (16). Focal uptake can involve a single site (unifocal), two sites (bifocal), or multiple sites (multifocal) (**Figure 1**) (1, 12, 21, 55). There has been controversy regarding the interpretation of PET positivity for bone marrow involvement in cases with diffuse bone marrow uptake on scans. Al-Sabbagh et al. found that none of the patients displaying diffuse bone marrow changes on PET-CT had a positive bone marrow biopsy, and conversely, none of the patients with a positive bone marrow biopsy exhibited diffuse bone marrow uptake on PET without a focal lesion (18). In contrast, another study reported that diffuse bone marrow uptake on PET-CT scans, conducted for patients with aggressive NHL, was frequently associated with a positive bone marrow biopsy (56). The pattern of bone marrow involvement in DLBCL is more commonly reported in a focal pattern on PET-CT as opposed to diffusely increased bone marrow uptake (12). The FDG uptake on PET-CT scans can vary in bone marrow involvement by different types of lymphomas, other than DLBCL (4, 40).

## 5 PET-CT versus bone marrow biopsy for detecting bone marrow involvement in DLBCL

In recent years, an ongoing debate has been ongoing regarding the potential replacement of bone marrow biopsy by PET-CT for evaluating bone marrow involvement in DLBCL. Several studies have examined the accuracy of each modality (**Table 4**).

**Table 4:** Comparing the ability of PET-CT and bone marrow biopsy in bone marrow involvement detection.

Reference	PET-CT	Bone marrow biopsy
Pelosi et al. – 2011 (50)	Sensitivity: 69% Specificity: 99.2% Accuracy: 91.4% PPV: 96.8% NPV: 90.2%	Sensitivity: 59.8% Specificity: 100% Accuracy: 89.6% PPV: 100% NPV: 87.7%
Khan et al. – 2013 (4)	Sensitivity: 94% Specificity: 100% Accuracy: 98.5%	Sensitivity: 40% Specificity: 100% Accuracy: 84%
Adam et al. – 2014 (57)	Sensitivity: 88.7% (95% CI: 82.5 – 93.3%) Specificity: 99.8% (95% CI: 98.8 – 100%)	
Cortes-Romera et al. – 2014 (58)	Sensitivity: 95% Specificity: 86% Accuracy: 87% PPV: 54% NPV: 99%	
Alzahrani et al. – 2016 (21)	Sensitivity: 60% (95%CI: 49-70) Specificity: 79% (95% CI: 75-83) PPV: 36% (95%CI: 28-44) NPV: 91% (95%CI: 88-94%)	
El karak et al. – 2017 (16)	Sensitivity: 92% Specificity: 100%	Sensitivity: 38% Specificity: 100%
Vishnu et al. – 2017 (2)	Sensitivity: 86% (95% CI: 51.9 – 95.7%) Specificity: 86% (95%CI: 76 – 92%) PPV: 50% NPV: 98% Accuracy: 86%	
Yilmaz et al. – 2017 (17)	Sensitivity: 91.3% Specificity: 94.3% Accuracy: 94% PPV: 67.7% NPV: 98.8%	
Saiki et al. – 2018 (46)	Sensitivity: 38% Specificity: 87% PPV: 53% NPV: 79%	
Xiao-Xue et al. – 2019 (34)	Sensitivity: 80% Specificity: 90% Accuracy: 88.1%	
Alsabbagh et al. – 2020 (18)	Sensitivity: 96% Accuracy: 99% NPV: 98%	Sensitivity: 50% Accuracy: 87% NPV: 84%
Kaddu-Mulindwa et al. – 2021 (15)	Sensitivity: 84% (95%CI: 78 – 88%) Specificity: 100% (95%CI: 99 – 100%) NPV: 95% (95%CI: 93 – 97%) PPV: 100% (95%CI: 98 – 100%)	Sensitivity: 38% (95%CI: 32 – 45%) Specificity: 100% (95%CI: 99 – 100%) NPV: 84% (95%CI: 81 – 86%) PPV: 100% (95%CI: 96 – 100%)
Almaimani et al. – 2022 (3)	Sensitivity 14.10 – 100% (median: 77.4%) Specificity: 54 – 100% (median: 91.65%) PPV: 29 – 100% (median: 63.60%) NPV: 81 – 100% (median: 97%)	Sensitivity: 24 – 68.8% (median: 47%) Specificity: 100% PPV: 100% NPV: 63.2 – 91.1% (median: 80%)
Asif et al. – 2023 (8)	Sensitivity: 93.16% Specificity: 93.93% PPV: 88% NPV: 96.88% Overall accuracy: 93.84%	

### 5.1 Precision of PET-CT vs. bone marrow biopsy in detecting bone marrow involvement

Numerous studies, including a meta-analysis, consistently report high sensitivity, specificity, and accuracy of PET-CT in detecting bone marrow involvement when compared to bone marrow biopsy (1, 3, 4, 8, 12, 16, 18, 59, 60). The negative predictive value and specificity of PET-CT for bone marrow involvement detection are exceptionally high, ranging between 85-98% and nearly 100%, respectively (3, 16, 34, 40). Rare instances have been documented where PET-CT produced negative results for bone marrow involvement, while bone marrow biopsy came back positive (18, 57, 61). These compelling findings have encouraged some groups to advocate for omitting routine bone marrow biopsy if PET-CT scan is positive (12, 18). One study reported no false-positive results for bone marrow involvement detected by PET-CT (18). Kaddu-Mulindwa et al. highlighted the superior accuracy and sensitivity of PET-CT over bone marrow biopsy for detecting bone marrow involvement, with rates reaching approximately 84% versus 38%, respectively (15). Bone marrow biopsy may generate false-negative results, particularly in cases of focal disease distant from the iliac crest (4). Adams et al. reinforced the inferior sensitivity of bone marrow biopsy for bone marrow involvement detection, citing a histologically proven bone marrow involvement rate of around 13-17% in newly diagnosed DLBCL cases (23). Some argue that patients with negative or limited focal bone marrow involvement away from the iliac crest on PET-CT may not benefit from bone marrow biopsy, suggesting its elimination in such cases (4).Nevertheless, certain groups advocate for the continued use of bone marrow biopsy before initiating treatment due to the potential for a worse prognosis with biopsy-proven bone marrow involvement and the ability to detect involvement missed by PET-CT (14, 16, 21). In the majority of studies, the percentage of bone marrow involvement missed by PET-CT, which was concordant DLBCL in the bone marrow, was minimal. Alzahrani et al. reported only 1% of patients with negative PET-CT had a positive concordant bone marrow biopsy (21). Another study confirmed that PET-CT successfully detected all cases with bone marrow involvement by DLBCL (4). However, a separate study reported a false negativity rate of PET-CT scans reaching almost 15% compared to the standard bone marrow biopsy, with most cases attributed to microscopic disease (40). Substantial evidence supports the complementary role of PET-CT with bone marrow biopsy for detecting bone marrow involvement, particularly in cases displaying diffuse bone marrow uptake on PET-CT (4, 59).

### 5.2 Impact of bone marrow involvement detected by PET-CT or bone marrow biopsy on staging, management, and outcome

Detection of bone marrow involvement through PET-CT in the literature has led to upstaging the disease to stage IV in nearly 25% of cases, whereas bone marrow biopsy has not been commonly associated with significant changes in the disease stage (4, 12, 18). In a limited number of cases, an elevation in the NCCN-IPI risk score has been reported, but there is insufficient evidence to establish a substantial difference in the actual disease stage for most patients (23). Management adjustments based on PET-CT positivity were observed in cases that were upstaged. However, in various studies, positive bone marrow involvement detected by biopsy rarely resulted in changes to the treatment plan (52, 53). Histologically discordant bone marrow involvement, particularly with low-grade lymphoma, typically does not lead to major alterations in the therapy plan, with treatment often directed toward the more aggressive lymphoma type (20, 52, 53). Nevertheless, it may prompt modifications in the management plan, including the potential use of maintenance rituximab and lifelong follow-up appointments (21). The prognostic impact of bone marrow involvement, whether detected by PET-CT or bone marrow biopsy, remains argumentative. Some studies suggest that histologically proven concordant bone marrow involvement by DLBCL is associated with an inferior prognosis, particularly in terms of OS and/or PFS, along with an increased risk of CNS involvement by DLBCL and CNS relapse (2, 4, 18, 20, 21, 61, 62). Critics argue that bone marrow biopsy typically identifies extensive disease involvement, contributing to the inferior outcomes in these patients (12, 49, 63, 64). Other studies report minimal or no impact of biopsy-proven bone marrow involvement on prognosis or the risk of CNS involvement, especially in cases with discordant bone marrow involvement (62, 65). Regarding bone marrow involvement detected by PET-CT as an independent adverse risk factor in DLBCL patients, most studies do not support this claim, particularly in patients already categorized as stage IV DLBCL for non-bone marrow involvement related criteria, where bone marrow involvement detection by PET-CT does not contribute to their inferior prognosis (4, 18, 23). However, a small study by Berthet et al. suggests that PET-CT positivity for bone marrow involvement serves as an independent risk factor for poor prognosis in DLBCL cases (41). Bone marrow involvement detected by both PET-CT and bone marrow biopsy together has been associated with a worse outcome than involvement detected by either method alone (1, 7, 12). A study by Cerci et al. reported an event-free survival (EFS) of around 78% (95% CI: 63-88%) and OS of 87% (95% CI: 73-94%) in DLBCL patients with focal FDG uptake in the bone marrow on PET-CT (12). In contrast, significantly inferior outcomes were reported in patients with both focal bone marrow involvement on PET-CT and positive bone marrow biopsy, with EFS and OS at 46% and 57%, respectively (12). This finding is supported by other studies, suggesting that combining both biopsy and PET-CT for detecting bone marrow involvement may be justified (1, 7). One study highlighted that bone marrow involvement in DLBCL detected by any method (i.e., bone marrow biopsy or PET-CT) influences staging, IPI score, and prognosis adversely (8). Despite the increased accuracy achieved by combining both biopsy and PET-CT for bone marrow involvement detection, a false-negative result does not completely rule out the presence of disease in the bone marrow (15).

### 5.3 Indicators elevating pretest probability for bone marrow involvement in DLBCL

**Figure 2:**
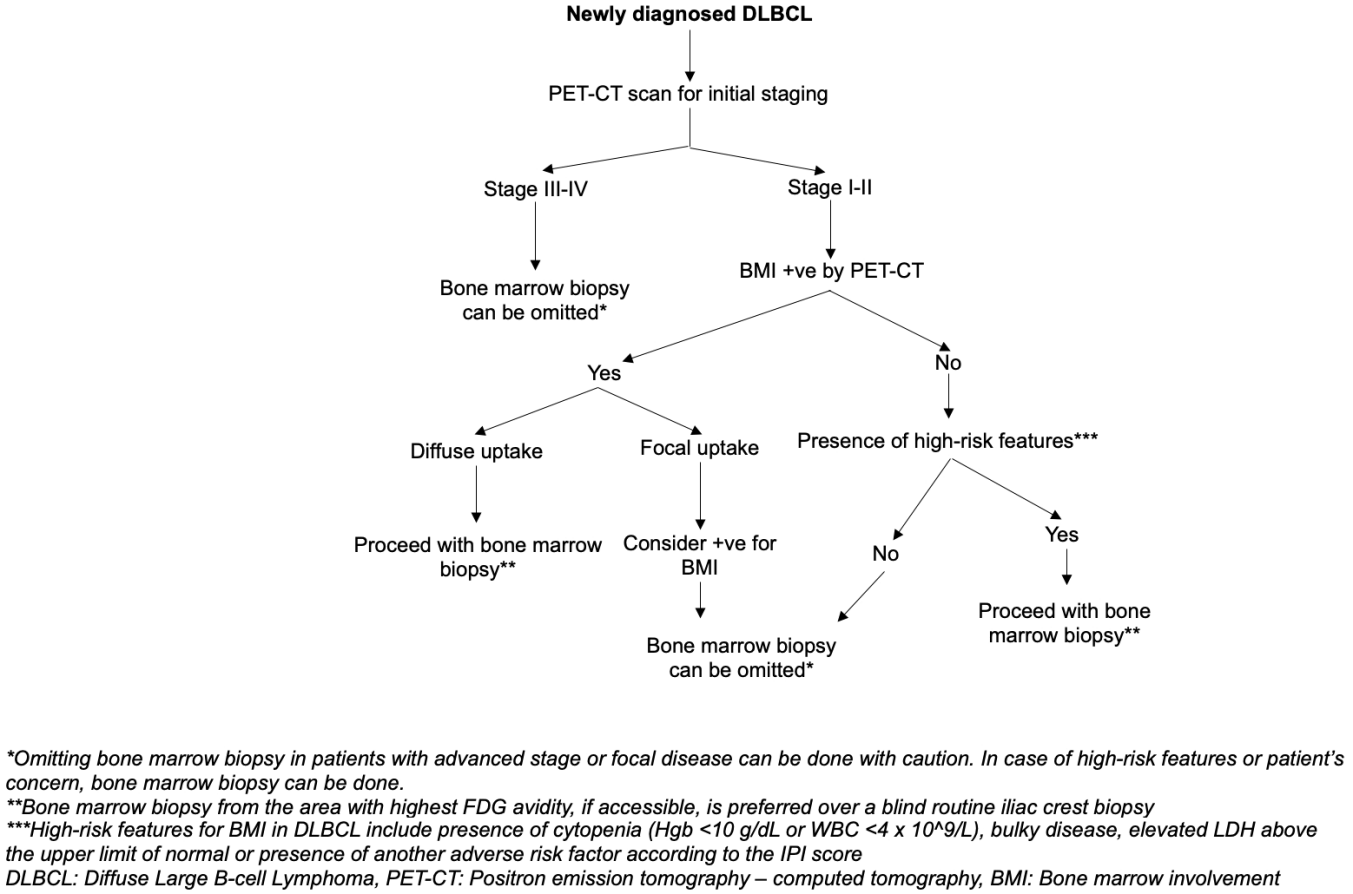
Suggested algorithm on the approach to bone marrow involvement assessment to identify the need for bone marrow biopsy in DLBCL staging.

Characteristic findings are frequently seen in DLBCL patients with bone marrow involvement. These findings can be used as markers to suggest the presence of bone marrow involvement and help guide the selection of diagnostic tools for the assessment of bone marrow involvement. Noteworthy features include the presence of cytopenia (i.e., Hemoglobin less than 10 g/dL, WBC less than 4 x 10^9/L) and/or bulky disease (66). In cases where anemia, leukopenia, and bulky disease are absent, the negative predictive value (NPV) approaches almost 99.2% (66). Additionally, an elevated LDH level above the upper limit of normal and the presence of adverse factors in the IPI score, excluding bone marrow involvement, should be considered as factors suggestive of bone marrow involvement (15). We suggest an algorithm designed to improve the accuracy of detecting bone marrow involvement in newly diagnosed DLBCL, as illustrated in **Figure 2**. However, the reliability and efficiency of this algorithm need to be thoroughly assessed through further studies.

## 6 Future insights

The absence of a definitive “gold standard” for bone marrow involvement detection in lymphoma persists. Efforts to enhance non-invasive techniques in identifying bone marrow involvement in aggressive lymphomas have been ongoing. PET-based radiomics is one intriguing technique being investigated; it combines PET imaging with radiomics, an approach that focuses on data extraction and the analysis of features from a large volume of images to uncover radiological patterns seen in the disease of interest that are frequently overlooked by conventional techniques, forecast treatment response, and acquire a deeper understanding of disease characteristics (67, 68). In a study by Filippi et al., 17 papers were reviewed, with 9 focusing on Non-Hodgkin lymphomas. These studies used multiple radiomic characteristics from baseline PET-CT scans to create machine learning-derived models. The models showed excellent results in predicting outcomes, especially the 2-year EFS in lymphomas. These results contribute to prognostication by highlighting the biological diversity and three-dimensional nature of lesions. Nevertheless, additional investigation, including validated prospective studies, is necessary to confirm the utility of PET-radiomics in the clinical practice (67). An alternative approach showing promise involves combining PET with MRI instead of CT. This innovative method holds potential as a tool that could replace the need for bone marrow biopsy, offering patients relief from the discomfort and pain that accompanies the biopsy (3, 16, 34).

## 7 Conclusion

The presence of bone marrow involvement has a significant impact on the prognosis and treatment of patients with DLBCL. While bone marrow biopsy has traditionally been considered the gold standard for evaluating such involvement, its use is accompanied by limitations and complications. The development and advancements in PET-CT and its precise capability to detect both nodal and extra-nodal involvement in DLBCL raise the possibility of precluding the need for bone marrow biopsy in this context. Although the histological examination of the bone marrow in DLBCL can have an impact on prognostication and can differentiate between concordant and discordant lymphoma cells in the bone marrow, yet the impact on the management of these patients is generally minimal, especially in patients classified as advanced stage by imaging. Both tools possess value but may not be universally necessary. Further cohort studies are needed to assess the validity of this statement and the applicability of the new advancements in the field of nuclear medicine to be standardized as the diagnostic tool for bone marrow involvement detection in DLBCL.

## Author contributions

RA: Conceptualization, Data curation, Formal analysis, Investigation, Methodology, Visualization, Writing – original draft, Writing – review & editing. RF: Writing – review & editing. AhA: Writing – review & editing. AbA: Supervision, Writing – review & editing. MK-D: Writing – review & editing. MA: Supervision, Writing – review & editing.
